# Kid-Sized Dentistry: A Pioneering First-of-Its-Kind Study on Customizing Dental Tools and Technology for Pediatric Care

**DOI:** 10.7759/cureus.69860

**Published:** 2024-09-21

**Authors:** Saurabh Tiwari, Debapriya Pradhan, Nikita Saini, Ankit Dhimole, Chirag Agrawal, Raunak Yadav, Aishwarya Jethi, Khushboo Singh, Kartik Sinha, Devika Agrawal

**Affiliations:** 1 Department of Pedodontics and Preventive Dentistry, Hitkarini Dental College and Hospital, Jabalpur, IND; 2 Department of Oral Medicine and Radiology, Hitkarini Dental College and Hospital, Jabalpur, IND; 3 Department of Pediatric Dentistry, Aarogya Dental and Skin Clinic, Udaipur, IND; 4 Department of Pedodontics and Preventive Dentistry, Pacific Dental College and Research Centre, Udaipur, IND

**Keywords:** children, dental practice, dental practice management, dental technology, dental tools, dentistry for kids, pediatric dentist, pediatric dentistry, pediatric instruments

## Abstract

Introduction

At the core of pediatric dentistry lies a profound understanding of child development, behavior management, and tailored treatment modalities. Similarly, tailoring dental tools and technology to address the distinct requirements of children is an essential aspect of pediatric dental practice. Kid-sized dentistry is the customization of dental tools and technology to effectively meet the unique needs of children. This study is the first to comprehensively explore all dimensions of resizing dental tools and technology for children, synthesizing scattered literature on the topic into a unified analysis, a trailblazing contribution to the field. Therefore, the aim of this article is to address these research gaps by conducting both a comprehensive analysis and a cross-sectional study.

Methods

To gather information for the subject, we conducted searches using both free-text terms and specific MeSH terms. We searched for articles published in English up to July 2024 across various electronic databases. Utilizing keywords, we initially obtained approximately 25-30 relevant literature studies, from which we selected 15-20 which were more topic-specific. We conducted an original survey to assess the knowledge, attitude, and practices of pediatric and general dentists regarding kid-sized dentistry in the Jabalpur zone. This survey employed a validated, structured questionnaire designed to collect data comprehensively.

Results

Despite high awareness, there remains a gap in the actual use of these tools and technology, particularly among general dentists.

Conclusion

In the realm of dentistry, every specialty brings a unique lens through which patient care is viewed. Within this mosaic, the pediatric dentist offers a particularly invaluable perspective, one that is grounded in the delicate nuances of treating children. Pediatric dentistry, in particular, focuses on the specialized needs of children, with an emphasis on understanding child development, behavior management, and customized treatment approaches. Central to this field is the adaptation of dental tools and technologies to suit pediatric patients. Insights from pediatric clinicians have contributed to advancements in dental equipment, improving its suitability for children. Increasing awareness of these innovations among both general and pediatric dentists can enhance this progress. This study underscores the need for ongoing education and advocacy to ensure all dental professionals have access to the best tools for treating young patients.

## Introduction

Pediatric patients cannot be viewed as miniature versions of grown-ups; their physiology and anatomy are distinct. To provide safe and effective care, it is crucial for dentists to grasp these physiological and anatomical disparities [1]. These dissimilarities between children and adults extend beyond their bodies to include variations in their behavior too [2]. Child’s constantly evolving cognition and psychology are more vulnerable to being influenced and have unique susceptibilities to form strong opinions. Therefore, the impact of dental treatment on a child can resonate throughout their lifetime [3]. Also acknowledging the physiological, anatomical, and psychological distinctions between children and adults is fundamental to delivering tailored oral health care to pediatric patients, effectively addressing their specific needs.

In pediatric dentistry, a key challenge is fostering a positive attitude toward dental care in children, starting with the selection of appropriately sized instruments and technology. Everything from dental tools or toothbrushes to advanced technologies like radiographic exposures is carefully "kid-sized" for a reason. Tailoring these essentials to fit children's unique needs ensures treatments are not only safe and effective but also comfortable. This thoughtful approach makes a big difference in delivering the best possible care for our young patients. Any discomfort, no matter how minor, can significantly affect a child's perception. Children are particularly sensitive to pain or discomfort, and a negative experience, no matter how small, can lead to fear, anxiety, or reluctance to seek dental care in the future. This is why it's crucial to minimize any potential discomfort during dental procedures, using gentle techniques and child-friendly approaches to create a positive and reassuring experience. So, we need to discard previous approaches like adult tools being adapted for use on children despite limitations in efficiency and precision due to their incompatibility with smaller oral cavities. Ensuring comfort not only helps in the immediate treatment but also plays a vital role in fostering lifelong trust in dental care.

“Kid-sized dentistry” is the adaptation of dental tools and technology to precisely fit the specialized needs of children. In particular, there is no published research covering all facets of kid-sized dentistry and no comprehensive analysis addressing its significance.

Significance of kid-sized dentistry

The American Academy of Pediatric Dentistry (AAPD) acknowledges that patient safety is a crucial aspect of providing quality oral health care for infants, children, adolescents, and individuals with special healthcare needs [4]. Pediatric patients exhibit a developing dentition and varying levels of compliance during dental procedures. Consequently, pediatric dental tools and technology are essential for providing effective and safe dental care to this demographic. They are meticulously designed to be atraumatic, non-intimidating, and highly efficient, ensuring a positive clinical experience for young patients. Categorization of the same can be done as:

Pediatric Clinical Setup 

Pediatric dental chair: The dental chair is a key element in the dental environment. The attractiveness of a pediatric dental chair aids in reducing fear and anxiety in children during dental visits. Its compact design offers several benefits, including adjustable headrests, optimal child positioning, close proximity to the child, easy access to the spittoon, appropriately sized instrument trays within reach of the pedodontist, and the facilitation of four-handed dentistry. These advantages make them more suitable than traditional adult dental chairs for treating young patients. Adult dental chairs are not ergonomically optimized for child care, which can decrease the efficiency of pediatric dentists and increase the risk of work-related musculoskeletal disorders [5].

Padded/papoose boards: Certain pediatric dental chairs incorporate additional design elements such as padded boards and papoose boards, which are frequently used to immobilize children during dental procedures. In this setup, the child is positioned on a flat board, and wide fabric straps are securely wrapped around the upper body, midsection, and legs. These restraints can be swiftly applied to prevent the kid from struggling or resisting treatment, thereby safeguarding the kid from potential injury [6].

Pediatric patient drapes: Pediatric patient drapes should be used for children not only because they are visually appealing but also because they provide effective protection against contaminants. When adult drapes are used on child patients, they often fit too loosely, especially around the neck, failing to offer adequate coverage and protection.

Pediatric Radiology

Image gently campaign: Radiographs are crucial for diagnosing and monitoring oral health issues in infants, children, adolescents, and individuals with special needs. They aid in evaluating trauma and tracking dental development and treatment progress. According to American Dental Association/Food and Drug Administration guidelines, the timing of initial radiographs should be based on individual's circumstances and should not be used for disease detection before a clinical examination [7]. The image gently in dentistry campaign aims to improve pediatric maxillofacial imaging safety by educating dental professionals and parents about radiation safety best practices and kid-sizing the exposure [8]. Digital intra-oral radiography significantly reduces radiation exposure compared to traditional film-based methods [9].

Pediatric lead apron, thyroid collar, radiographic films, and RadioVisioGraphy (RVG) sensors: Ergonomic design of the apron and collar must be used to ensure a proper fit and effective radiation protection for children. Additionally, using bright colors for these protective garments can help in managing child patients by creating a more comforting and engaging environment. Pediatric RVG sensors (Size 0) should be utilized. In imaging software, even the exposure layout can be changed to a pediatric specific mode. For imaging anterior teeth in children, size 1 films (24 x 40 mm) are preferred due to their narrower shape, which captures detailed images with minimal discomfort. For posterior teeth, size 0 films (22 x 35 mm) are recommended as their smaller size reduces the risk of triggering the gag reflex, enhancing both patient comfort and cooperation [10].

*Pediatric Diagnostic and Isolation Armamentarium* 

Miniature, colorful diagnostic and isolation armamentariums are used to examine/visualize hard-to-reach areas inside the child’s smaller oral cavity, aiding in treatment, ensuring easier access, better isolation, preventing trauma, and making the procedure appear stress-free and approachable for the child. The following are available in miniature sizes: mouth mirrors, tweezers, spoon excavators, intra-oral mirrors, cheek retractors, impression trays, cotton rolls, rubber dam instrument kits, and bite blocks. Loose primary teeth can be dislodged and potentially swallowed or aspirated when using any type of mouth prop. To prevent this, it is advisable to examine the dentition as a precaution before employing a mouth prop. The AAPD guidelines on Behavior Guidance specify that using a mouth prop on a cooperative child is not categorized as stabilization. However, when used on an uncooperative child, it is considered protective stabilization and necessitates obtaining informed consent. Pediatric dentistry employs a variety of mouth props like McKesson style, Molt adjustable, prop with extraoral handle, disposable foam, and others [11]. The dry shield isolation system is a new alternative to the rubber dam isolation in pediatric dentistry. Developed in the United States, it resembles the isolate system isolation but differs in that it lacks illumination and is autoclavable. The system includes a bite block, and cheek and tongue retractors connected to high-volume suction, offering dual functions of retraction and suction. Additionally, the bite block aids in mouth opening and stabilization. Another advantage is its ability to retract and isolate one side of the oral cavity simultaneously, providing access to both upper and lower quadrants at once [12].

Pediatric Endodontics

Mini head airotor and short shank burs make treatment easier due to easier accessibility in the kid's mouth. The extended length of adult files presents challenges in kids, increasing the risk of breakage and mouth fatigue compared to shorter files. Therefore, it is advisable to use shorter hand files (18mm and 21mm) in their oral cavity. The major limitations while using adult rotary files in primary teeth are the thinner curved roots with ribbon-shaped morphology increasing the chance of lateral perforation. This creates the requirement for pediatric endodontic rotary files [13].

Pediatric Prosthodontics

Preformed crowns of diverse materials, shapes, and sizes are employed in pediatric dentistry to ensure better adaptation and to minimize the time taken for chairside work, impression taking, and laboratory fabrication.

Preventive Dentistry

Preformed band and space maintainers are also employed. Toothpaste is tailored to meet the fluoride needs and creative modified toothbrush designs are available to not only improve a kid's hand grasp of the same but to also allow it to fit the anatomy of a child's oral cavity, ensuring effective cleaning beyond just aesthetic appeal.

Pediatric Oral Surgery

Many dentists opt to use the same surgical instruments for both pediatric and adult patients. However, most pediatric dentists and oral and maxillofacial surgeons prefer smaller pediatric extraction forceps for several reasons, like their reduced size facilitates easier placement in the smaller oral cavity of pediatric patients, the smaller pediatric forceps are more easily concealed by the operator's hand and the smaller working ends (beaks) closely conform to the anatomy of primary teeth. Choosing the appropriate instrument also depends on unique considerations for children and adolescents. Trauma/injury can be caused if kid-sizing is ignored, for example, cow horn mandibular forceps are not employed for primary teeth as they pose a high risk of injuring developing premolars. Care must also be taken when using elevators and forceps near large restorations, such as chrome crowns, and adjacent to erupting single-rooted teeth that may dislodge easily with minimal force [14].

Rainbow Presidential Pediatric Forceps (F150SR, F151SR: Hu-Friedy Rainbow Presidential Pediatric Forceps series, among other pedo series' forceps of the same brand) are designed with a vibrant array of colors to alleviate child anxiety and foster a positive patient experience, in addition to being kid-sized. Another example of kid-sizing dental instruments is a novel extraction forcep which includes precautions to prevent accidental swallowing or aspiration of extracted teeth in children. The forceps feature short beaks that fit snugly around the crown and root of the tooth. When pressure is applied, the beaks move apically, allowing the tooth to move incisally into a specially designed chamber within the forceps. This chamber securely holds the tooth in place until the forceps are removed from the oral cavity. Thus, the design of the forceps aims to enhance tooth extraction safety [15].

This study is the first to comprehensively explore all dimensions of resizing dental tools and technology for children, synthesizing scattered literature on the topic into a unified analysis, a trailblazing contribution to the field. Therefore, the aim of this article is to address these research gaps by conducting both a comprehensive analysis and a cross-sectional study.

## Materials and methods

An original cross-sectional study was conducted to evaluate the knowledge, attitude, and practices of pediatric and general dentists in Jabalpur zone regarding kid-sized dentistry.

Study design

The survey utilized a validated, structured, self-explanatory, close-ended questionnaire to gather comprehensive data. A total of 15 questions, written in English, were prepared and emailed to five experts for content validation using a five-point Likert scale. The Aiken index was calculated for each question to determine its relevance, with only those scoring ≥0.6 being included in the final questionnaire. Reliability was assessed using Cronbach’s α, with values ranging between 0.78 and 0.86 and a median of 0.82 deemed acceptable. These questions are presented in the tables within the results section. The questionnaire also included queries about personal details, such as profession/specialization and years of practice as it was needed for an accurate statistical analysis. Out of 15 questions, 5 were designed to assess knowledge, 5 focused on attitudes, and 5 evaluated practices.

Inclusion criteria

Only the participants who held either a Bachelor of Dental Surgery (BDS) degree or a Master’s degree in Pediatric and Preventive Dentistry were included in the study. It was made sure that the participants were practicing within the Jabalpur zone to ensure regional relevance and consistency in practice settings. They were required to complete and submit the questionnaires on the same day they received them, ensuring timely and accurate data collection. Only active practitioners with current engagement in clinical dental practice were included to ensure the relevance of their responses to the study objectives.

Exclusion criteria

Participants lacking either a Bachelor of Dental Surgery (BDS) or a Master’s degree in Pediatric and Preventive Dentistry were excluded from the study. To ensure only qualified professionals were involved, individuals still in the process of earning their undergraduate or postgraduate dental degrees were also excluded. Dentists specializing in fields other than Pediatric and Preventive Dentistry were excluded to preserve the study's focus and consistency. Additionally, those practicing outside the Jabalpur zone were not considered to maintain regional relevance. Participants who did not submit their completed questionnaires on the same day they were distributed were excluded to uphold the study’s standards for prompt and accurate data collection. Finally, dentists who were not actively engaged in clinical practice were excluded to ensure that the study data accurately reflects current professional experiences.

Statistical analysis

Using the sample size formula with a power of 80% and zα2 of 1.96, and assuming an 8% improvement in training, we selected a total of 108 participants, with 54 pediatric dentists and 54 general dentists. Now, the responses were statistically analyzed using IBM SPSS Statistics for Windows, Version 22 (Released 2013; IBM Corp., Armonk, New York, United States), applying the test of proportions and the chi-square test for evaluation.

## Results

All the results regarding the KAP (Knowledge, Attitude and Practices) of study participants were tabulated and the total percentage was calculated. The distribution of perceptions regarding dentists' knowledge of kid-sized dentistry is illustrated in Table 1.

**Table 1:** Knowledge-based questions and their analysis

S. No.	Question	Qualification	Options	
			Yes in N(%)	No in N(%)
1	Are you aware of the recent trend of “kid–sized dentistry”?	General practitioner	30 (57.4%)	23 (42.6%)
		Pediatric dentist	38 (72.2%)	15 (27.8%)
2	Are you aware of the “Image Gently Campaign” for kids in dentistry?	General practitioner	13 (24.1%)	40 (75.9%)
		Pediatric dentist	25 (46.3%)	28 (53.7%)
3	Are you aware that customized isolation tools are available to be used in pediatric dental practice such as Pediatric rubber dam kit, pedo cotton rolls, dry shield, etc.?	General practitioner	52 (96.3%)	1 (3.7%)
		Pediatric dentist	54 (100.0%)	0 (0.0%)
4	Are you aware that pediatric dental extraction forceps when used in kids provide a more positive patient experience as compared to adult forceps as they require less force, reducing the risk of tissue damage and broken crowns?	General practitioner	48 (88.9%)	5 (11.1%)
		Pediatric dentist	52 (96.3%)	1 (3.7%)
5	Are you aware that there are increased chances of root perforation while using adult rotary files instead of Pediatric rotary files during primary tooth pulpectomy procedure?	General practitioner	44 (83.3%)	9 (16.7%)
		Pediatric dentist	46 (87.0%)	7 (13.0%)

The dentists' attitudes toward the need for customizing dental technology to meet children's needs are shown in Table 2.

**Table 2:** Attitude-based questions and their analysis

Question	General practitioner (Yes) N(%)	General practitioner (No) N(%)	Pediatric dentist (Yes) N(%)	Pediatric dentist (No) N(%)
1) Do you think it is unsafe to use longer-length hand files while performing pulpectomy in pediatric patients?	46 (85.2%)	8 (14.8%)	48 (88.9%)	6 (11.1%)
2) Do you think that there are minimal chances of iatrogenic injuries when kid-sized instruments are used for the treatment of pediatric patients?	48 (88.9%)	6 (11.1%)	53 (98.1%)	1 (1.9%)
3) Do you think it is necessary to kid-size the exposure time when using a radiograph during pediatric dental procedures?	49 (90.7%)	5 (9.3%)	54 (100.0%)	0 (0.0%)
4) Do you think using an airotor with a small head size improves visibility and accessibility in children?	54 (100.0%)	0 (0.0%)	54 (100.0%)	0 (0.0%)
5) Do you feel the need of using instruments with smaller mouthpieces and customized smaller biteblocks for comfortable experience of kids during their dental treatment?	52 (96.3%)	2 (3.7%)	54 (100.0%)	0 (0.0%)

 The prevailing kid-sized dentistry practice patterns among dentists when treating pediatric patients are outlined in Table 3.

**Table 3:** Practice-based questions and their analysis

S.no	Question	Qualification	Options	
			Yes in N(%)	No in N(%)
1	Do you use kid-sized dental instruments in your clinical practice while examining and treating patients?	General practitioner	25 (46.3%)	29 (53.7%)
		Pediatric dentist	54 (74.1%)	14 (25.9%)
2	Do you use pedo sensors when taking radiographs during pediatric dental procedures?	General practitioner	21 (38.9%)	33 (61.1%)
		Pediatric dentist	32 (59.3%)	22 (40.7%)
3	Do you use a mini head airotor for efficient treatment in pediatric patients?	General practitioner	25 (46.3%)	53 (98.1%)
		Pediatric dentist	36 (66.7%)	18 (33.3%)
4	Do you attend any pediatric dental educational program to acquire awareness regarding the latest tools for pediatric dentistry?	General practitioner	27 (50%)	27 (50%)
		Pediatric dentist	34 (63%)	20 (37%)
5	Do you promote the use of customized dental instruments for treating pediatric patients among other dental professionals?	General practitioner	37 (68.5%)	54 (31.5%)
		Pediatric dentist	44 (81.5%)	10 (18.5%)

**Figure 1:**
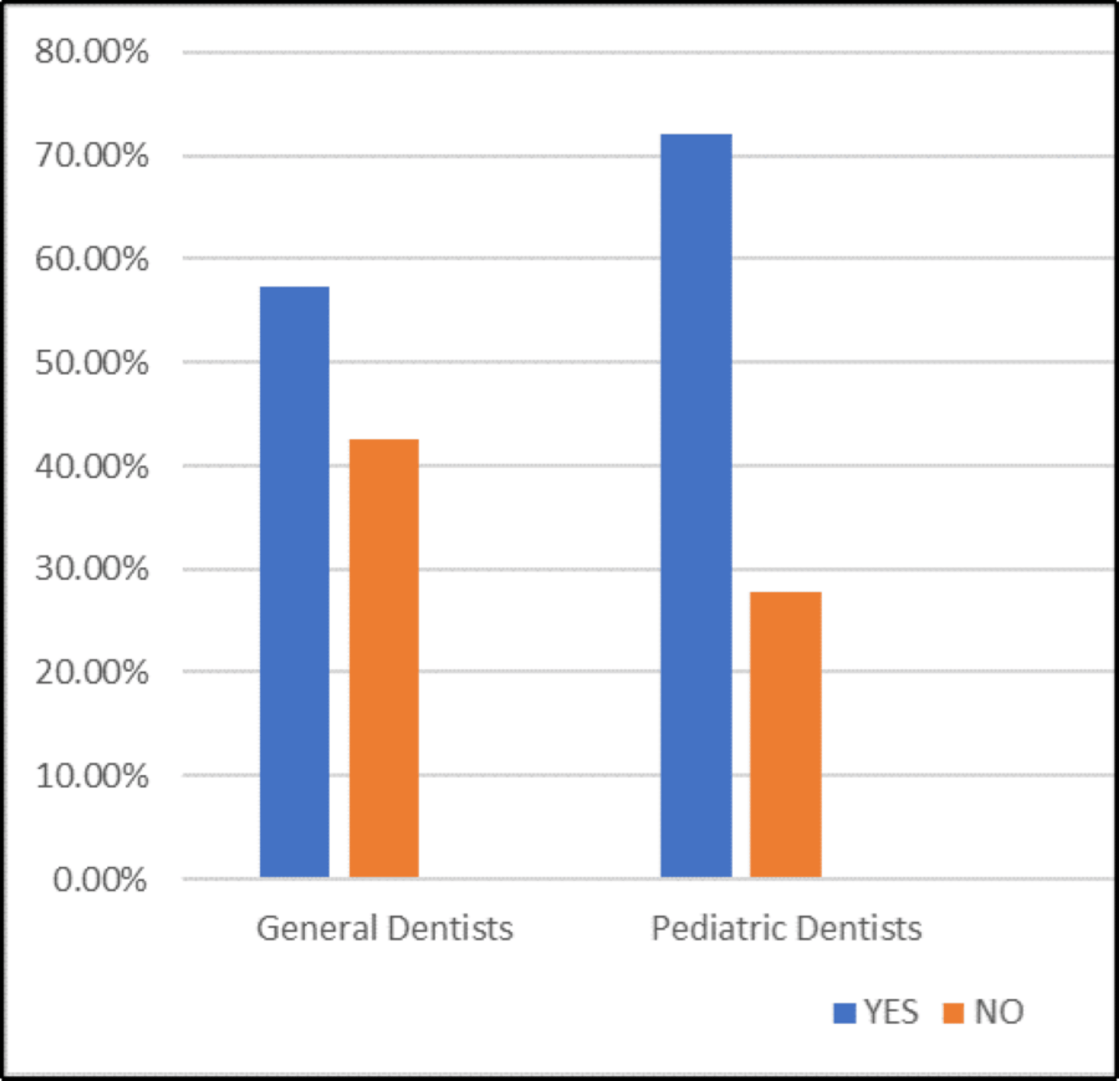
Awareness levels of the recent trend of “kid-sizing dental tools and technology” among study participants

Awareness levels of the recent trend of “Kid-Sizing Dental Tools and Technology” among study participants are depicted in Figure 1. The graph shows a higher level of awareness about kid-sized dental tools among pediatric dentists compared to general dentists. Around 75% of pediatric dentists are familiar with these tools, while general dentists show a more balanced split, with approximately 55% being aware. This stark difference underscores the increased relevance and familiarity of pediatric-specific tools within pediatric dental practices compared to general dentistry, likely due to their routine engagement with younger patients. Given the importance of appropriately sized tools for child patients, it is crucial that general dentists and the unaware population of pediatric dentists, who also treat children, improve their awareness and adapt their practices accordingly.

**Figure 2:**
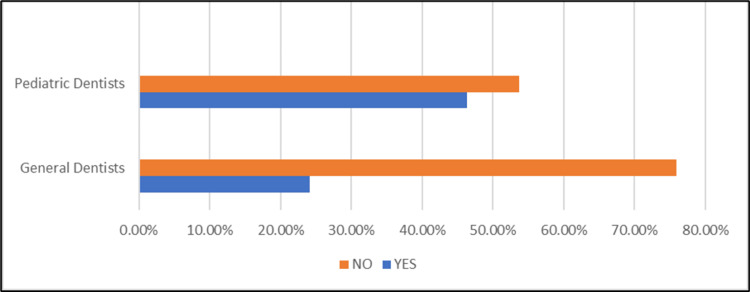
Awareness levels of the image gently campaign among study participants

The awareness levels for "image gently campaign of radiation exposure" among the study participants are shown in Figure 2. The graph shows low awareness of the campaign among both general and pediatric dentists. About 75% of general dentists and 60% of pediatric dentists are unfamiliar with the campaign, underscoring the need for greater promotion of its safety guidelines. 

## Discussion

Kid-sized dentistry, which we defined as the adaptation of dental tools and technology to precisely fit the specialized needs of children, is important for several reasons.

Comfort and safety

Equipment designed specifically for children is better suited to their smaller mouths and delicate tissues, reducing discomfort and the risk of injury during dental procedures.

Effectiveness

Customizing dental technology for children ensures that treatments are more effective, as tools tailored to their size and needs can enhance the precision and quality of dental care.

Reduced anxiety

Child-friendly dental tools and environments help alleviate fear and anxiety in young patients, making dental visits more pleasant and encouraging regular dental check-ups.

Improved compliance

When children feel comfortable and less intimidated by dental procedures, they are more likely to cooperate, which facilitates smoother and more efficient treatments.

Positive long-term attitudes

Pleasant early dental experiences can foster good oral hygiene habits and a positive attitude toward dental care, leading to better oral health throughout their lives.

Specialized treatment needs

Children often require different dental treatments than adults, such as space maintainers, which necessitate specialized tools and techniques to address their unique dental development stages.

**Figure 3:**
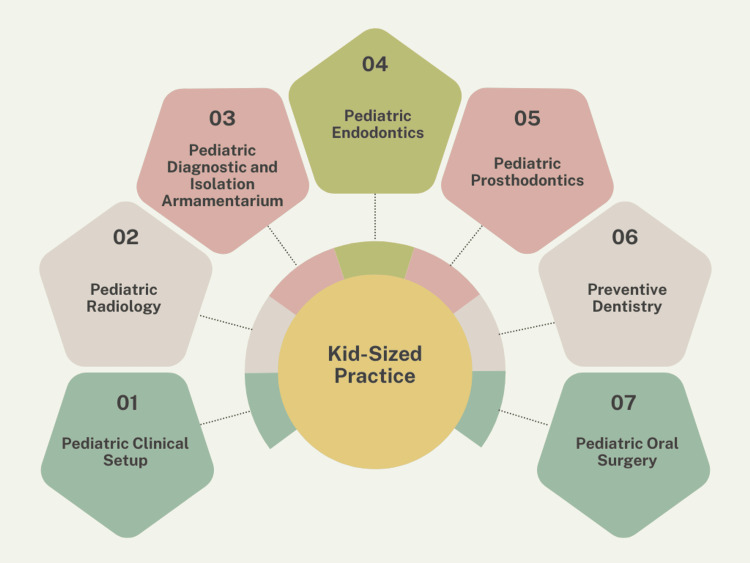
Kid-sizing of dental tools and technology seen in various categories This has been categorized for the first time (image credit: Tiwari S).

Kid sizing of dental tools and technology can be seen in various categories as shown in Figure 3.

This groundbreaking study is the first of its kind to evaluate the understanding and significance of kid-sized dental tools and technology in pediatric dental practice. The level of awareness among study participants about the kid-sizing of dental tools and technology is illustrated in Figure 1.

High-speed handpieces come in various head sizes, ranging from 9.8 x 8.5 mm to 14.5 x 13 mm. Miniature head-size handpieces are specifically designed for pediatric patients and adults with limited mouth opening, enhancing visibility during dental procedures [16]. Our findings reveal a surprising gap: while all general dentists knew that using an airotor with a small head size improves visibility and accessibility in children, only 46.3% actually incorporate these specialized tools and mini head airotors into their clinical practice for treating pediatric patients. An impressive population of pediatric dentists (98.1%) believe that the use of kid-sized instruments significantly minimizes the risk of iatrogenic injuries during pediatric treatments. Furthermore, every single one of them (100%) underscored the essential need for instruments with smaller mouthpieces and customized bite blocks to ensure a comfortable and positive experience for children during dental procedures.

In a study conducted by Jeevanandan et al., the use of pediatric-specific rotary files was shown to significantly improve the quality of root canal obturation and preparation [17]. Building on this research, our study revealed that 83.3% of general dentists and 87% of pediatric dentists acknowledged the heightened risk of root perforation when using adult rotary files instead of pediatric ones during primary tooth pulpectomy procedures. These findings align with research by Alowi et al., which indicated that 55.6% of dentists prefer using dedicated rotary files for primary teeth root canal preparation due to their enhanced accessibility and precision [18].

Our survey revealed that a significant proportion of both general and pediatric dentists are aware of the availability of customized isolation tools for pediatric dental practice, such as pediatric rubber dam kits, pedo cotton rolls, and dry shields. They also understand that using pediatric dental extraction forceps provides a more positive patient experience compared to adult forceps, as they require less force and reduce the risk of tissue damage and broken crowns. Additionally, they recognize the dangers of using longer hand files during pulpectomy procedures in a child. Despite this extensive knowledge, it is striking that 18.5% of pediatric dentists and 31.5% of general dentists have not fully embraced advocating for the use of customized dental instruments in pediatric patient care among their peers in the dental community.

It is important to reduce radiation exposure in children because they are more vulnerable than adults. Their bodies have a higher risk of developing cancer from radiation, and they have a longer time for any potential effects to show up later in life. Adult X-ray settings can result in unnecessarily large doses for pediatric patients. In 2007, the Society for Pediatric Radiology, along with several major organizations, founded the Alliance for Radiation Safety in Pediatric Imaging. More than 80 groups have now joined the Image Gently campaign to improve patient care and practices through education and awareness [19]. Supported by major organizations, the campaign emphasizes the fact that "one size does not fit all", thus it is important to kid-size pediatric radiographic procedures. It draws attention to the fact that X-rays should be customized to individual needs, using the fastest receptors, collimating the beam, always applying thyroid collars, adjusting exposure for children, and opting for cone-beam CT with dose reduction when appropriate [8].

It is encouraging that 90.7% of general dentists and all pedodontists understand the importance of adjusting exposure time for pediatric radiographs. However, the use of pediatric sensors during these procedures remains limited, with only 38.9% of general dentists and 59.3% of pediatric dentists incorporating them into their practice.

A concerning revelation is that half of the general dentists and 37% of pedodontists have not attended any pediatric dental educational programs to stay updated on the latest tools for pediatric dentistry. This gap raises questions about their commitment to ensuring safe and efficient pediatric practice. By focusing on kid-sized dentistry, dental professionals can provide higher-quality, safer, and more effective care tailored to the unique needs of their young patients.

Study limitation

Since it is a questionnaire-based study, response bias might have resulted from participants giving socially desirable answers. Another limitation is that it has been conducted in a smaller regional area. The study’s generalizability is therefore limited. To assess awareness on a broader scale, similar studies with larger geographic coverage are needed.

## Conclusions

The ongoing trend of "kid-sizing" dental tools and technology significantly impacts the evolution of pediatric dentistry, prioritizing easier and more efficient treatment by dentists while ensuring safety for children. Encouraging the use of customized dental instruments among dental professionals is crucial to ensure positive dental experiences for pediatric patients. This is particularly important considering children's smaller jaw size and unique anatomy. Dentists should actively participate in pediatric dental education programs to stay updated on the latest advancements in tools and technology designed specifically for pediatric dentistry.
